# Construction and investigation of a combined hypoxia and stemness index lncRNA-associated ceRNA regulatory network in lung adenocarcinoma

**DOI:** 10.1186/s12920-020-00816-8

**Published:** 2020-11-04

**Authors:** Lili Guo, Hongxia Li, Weiying Li, Junfang Tang

**Affiliations:** 1 Department of Medical Oncology, Beijing Tuberculosis and Thoracic Tumor Research Institute, Beijing Chest Hospital, Capital Medical University, No. 1 Beiguandajie, Tongzhou District, Beijing, 101149 China; 2 Department of Cellular and Molecular Biology, Beijing Tuberculosis and Thoracic Tumor Research Institute, Beijing Chest Hospital, Capital Medical University, Beijing, 101149 China

**Keywords:** Lung adenocarcinoma, Hypoxia, Stemness, ceRNA, Differentially expressed gene

## Abstract

Hypoxia and stemness are important factors in tumor progression. We aimed to explore the ncRNA classifier associated with hypoxia and stemness in lung adenocarcinoma (LUAD). We found that the prognosis of LUAD patients with high hypoxia and stemness index was worse than that of patients with low hypoxia and stemness index. RNA expression profiles of these two clusters were analyzed, and 6867 differentially expressed (DE) mRNAs were screened. Functional analysis showed that DE mRNAs were associated with cell cycle and DNA replication. Protein–protein interaction network analysis revealed 20 hub genes, among which CENPF, BUB1, BUB1B, KIF23 and TTK had significant influence on prognosis. In addition, 807 DE lncRNAs and 243 DE miRNAs were identified. CeRNA network analysis indicated that AC079160.1-miR-539-5p-CENPF may be an important regulatory axis that potentially regulates the progression of LUAD. The expression of AC079160.1 and CENPF were positively correlated with hypoxia and stemness index, while miR-539-5p expression level was negatively correlated with hypoxia and stemness index. Overall, we identified CENPF, BUB1, BUB1B, KIF23 and TTK as potentially key genes involved in regulating hypoxia-induced tumor cell stemness, and found that AC079160.1-miR-539-5p-CENPF axis may be involved in regulating hypoxia induced tumor cell stemness in LUAD.

## Introduction

Lung cancer is one of the malignant tumors with the highest morbidity and mortality in the world [1]. Lung adenocarcinoma (LUAD) is one of the common pathological types of lung cancer [2]. In recent years, the incidence of LUAD in many countries and regions has exceeded that of squamous cell carcinoma, becoming the most common pathological type of lung cancer [3]. The 5-year survival rate of LUAD is only about 15% [4]. The prognosis of LUAD patients is extremely unsatisfactory [4]. Therefore, the treatment of LUAD must start from the "root". Cancer stem cell theory proposes that in addition to ordinary tumor cells, there are a small number of tumor-propagating cells that can self-renew, continue to proliferate and differentiate [5, 6]. These cells are the seed of a poor prognosis such as tumor recurrence, metastasis and chemotherapy resistance [4, 5]. In recent years, cancer stem cells have been successfully isolated from more and more different types of cancer cells, which provides a solid basis for stem cell theory and makes cancer stem cells become a hot research topic in the field of cancer [7, 8].

Hypoxia is an important feature of microenvironment in solid tumor [9, 10]. During the occurrence and development of malignant tumors, due to the imbalance between the growth rate of tumor tissue and the oxygen supply capacity of the tissue, the solid tumor tissue survives in a special hypoxic microenvironment [9, 11]. Hypoxia is closely related to tumor invasion and metastasis [12]. In the hypoxic microenvironment, the tumor cells adapt to the environment to screen out the tumor cells with strong invasive ability, namely tumor stem cells [13]. By changing epigenetics and genetic stability, tumor stem cells with different clonal subpopulations, growth rates and degrees of chemotherapy resistance can be generated [14]. However, these factors have not meant considered for the treatment of LUAD because of the lack of effective biomarkers.

Most of the human genome is non-coding regions, which are transcribed to non-coding RNA (ncRNA). Although ncRNA cannot be translated into protein, it can affect human physiological and pathological processes by directly or indirectly regulating mRNA [15, 16]. In 2011, Salmena et al. proposed the competitive endogenous RNA (ceRNA) hypothesis [17]. The hypothesis holds that microRNA (miRNA) is the core element in the ceRNA network, while long non-coding RNA (lncRNA), etc., competes with ceRNA for one or more miRNA reaction elements to regulate the function of other RNAs [17, 18]. Studies have shown that ceRNA network plays an important regulatory role in the regulation of cell cycle and cell death in various malignant tumors such as lung cancer, affects tumor invasion and migration, thereby playing a critical role in the occurrence and development of tumors [19, 20]. NcRNA can be used as a potential target marker for early diagnosis, treatment and prognosis of tumors [21–23]. However, the relationship between LUAD hypoxic microenvironment, tumor stemness and ceRNA still needs further study.

The Cancer Genome Atlas (TCGA) is a publicly funded project that provides public cancer data sets to improve diagnostic methods, treatment standards, and ultimately prevent cancer [24]. In this study, the bioinformatics analysis method was used to analyze expression data of LUAD in the TCGA database, seeking to develop a hypoxic and stemness related ncRNA classifier to provide new ideas for the prognosis of LUAD.

## Methods

### Dataset

RNA sequencing data and corresponding clinical data of LUAD patients were downloaded from TCGA and GEO (GSE31210) databases. Hypoxia index was calculated using GSVA algorithm according to the hypoxia system-related metagene clusters [25, 26]. mRNA expression-based stemness index (mRNAsi) is an index that describes the similarity between cancer cells and stem cells, and it might be considered a quantitative form of cancer stem cells. The mRNAsi of LUAD cases were obtained from previous studies [27]. Unsupervised two-dimensional hierarchical clustering was applied to cluster the LUAD samples, and the similarity between the samples was evaluated using the Euclidean distance. Differences in prognosis among different clusters were analyzed by Kaplan–Meier curve and log-rank test. Meanwhile, miRNA sequencing data corresponding to clinical data of 447 patients were downloaded from TCGA database.

### Screening for differentially expressed (DE) mRNA, lncRNA and miRNA

Paired t-test was used to screen DE mRNA, lncRNA or miRNA between cluster 3 and cluster 4, and multiple P-value correction was performed for multiple tests. mRNA, lncRNA or miRNA with fold change (FC) > 1.5 and *P* < 0.05 were selected as differentially expressed genes.

### Functional characterization of DE mRNAs

The biological function of DE mRNAs was characterized by gene ontology (GO) terms and Kyoto Encyclopedia of Genes and Genomes (KEGG) pathway. The threshold of enrichment significance was *P* < 0.05. Protein‐protein interaction (PPI) network was constructed using Cytoscape software (https://www.cytoscape.org). The DE genes were ranked according to Maximal Clique Centrality (MCC) score, and the top 20 were identified as hub genes.

### Prognostic analysis

In order to evaluate the impact of hub genes expression on patient survival, we downloaded the mRNA expression data and clinical information from TCGA and Kaplan Meier-plotter databases, respectively. According to the gene expression level, patients were divided into high expression group and low expression group. The overall survival (OS) of patients with LUAD were assessed using Kaplan–Meier survival plot.

Kaplan–Meier curve and log-rank test was also performed to predict the effect of DE lncRNA and DE miRNA on prognosis. The overall hazard ratios (HRs) with 95% confidence interval (CI) were calculated to evaluate the prognostic role of differentially expressed lncRNA or miRNA on patients with LUAD.

To assess the effect of the copy number of DE gene on prognosis, cBioPortal database (https://www.cbioportal.org/) was applied to analysis the alteration frequency of gene. Patients were divided into altered group and unaltered group. The overall survival was assessed using Kaplan–Meier curve.

### Construction and correlation analysis of ceRNA network

Hub genes, DE lncRNAs and DE miRNAs that significantly affected prognosis were applied to construct the ceRNA network. StarBase (https://starbase.sysu.edu.cn/) was used to establish ceRNA network. Cytoscape software was used to visualize the ceRNA network. Correlation analysis of node expression in ceRNA network with hypoxia and stemness index was performed using R software program.

### Statistical analysis

Statistical analysis was performed using R software. Student’s *t*-test and one-way ANOVA were used to determine the statistical significance. *P* < 0.05 was considered as statistically significant difference.

## Results

### The effects of hypoxia and stemness on the survival of patients with LUAD

**Fig. 1:**
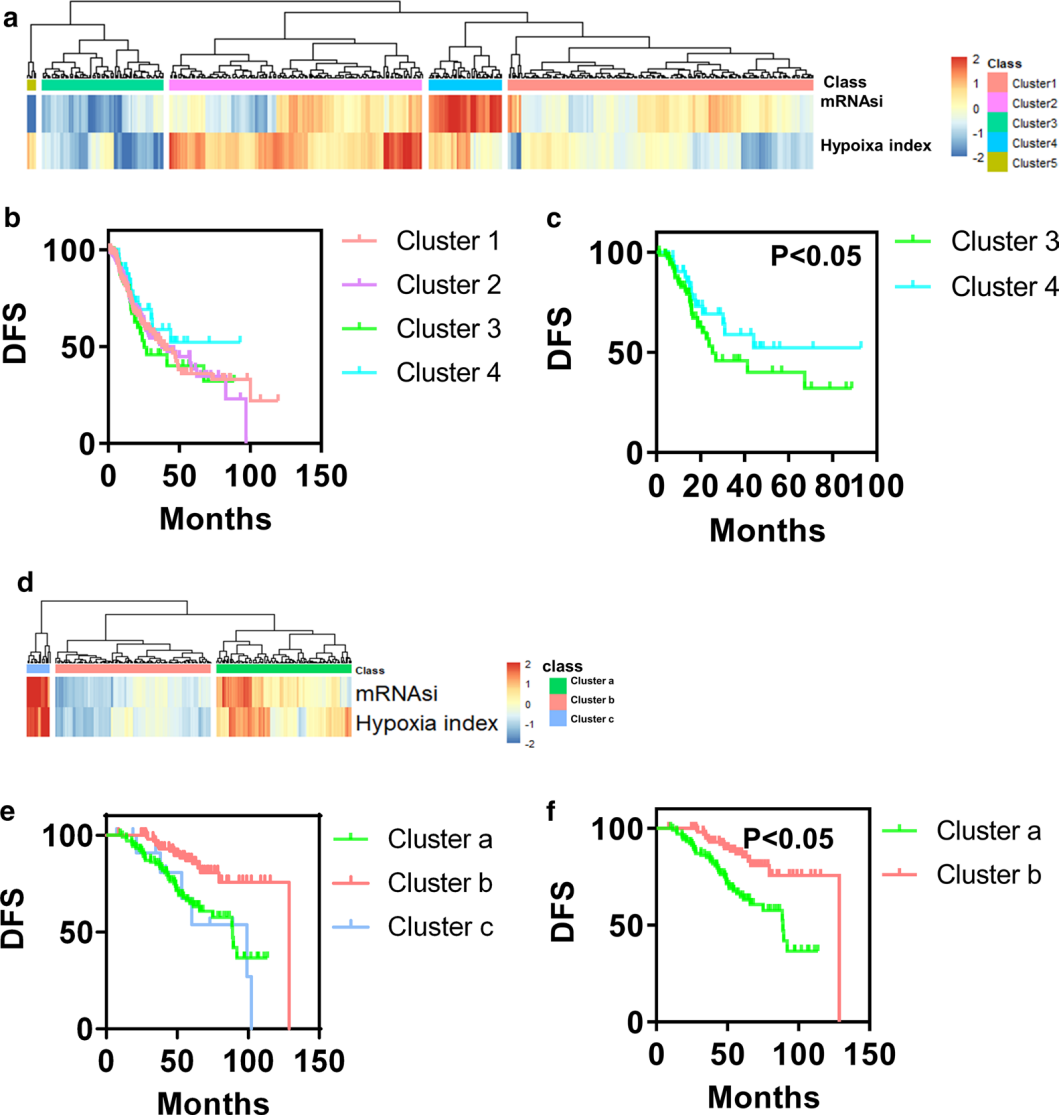
Cluster analysis and prognosis analysis of patients with LUAD. **a** Unsupervised two-dimensional hierarchical clustering was performed according to the hypoxia and stemness index of lung cancer patients in TCGA database. Cluster 1 (N = 177), cluster 2 (N = 152), cluster 3 (N = 68), cluster 4 (N = 44) and cluster 5 (n = 6). **b**, **c** Comparison of survival curves of 5 clusters. **d** Patients in GSE31210 were clustered based on hypoxia and stemness index using unsupervised two-dimensional hierarchical clustering method. Cluster a (N = 103), cluster b (N = 105) and cluster c (N = 13). **e**, **f** Prognosis analysis of patients in cluster a, b and c. mRNAsi, mRNA expression-based stemness index

To understand the effect of hypoxia and stemness on lung cancer progression, 447 patients (in TCGA database) were divided into 5 groups by unsupervised two-dimensional hierarchical clustering according to the hypoxia and stemness index: cluster 1 (N = 177), cluster 2 (N = 152), cluster 3 (N = 68), cluster 4 (N = 44) and cluster 5 (N = 6) (Fig. 1a). Then, we compared the differences in disease-free survival (DFS) among the different groups (Fig. 1b). The difference in DFS between cluster 3 and cluster 4 was significant (Fig. 1c), while the difference in DFS among other groups was not significant (Fig. 1b). Cluster analysis revealed that the stemness and hypoxia index of cluster 3 was lower, while the stemness and hypoxia index of cluster 4 was higher (Fig. 1a). In addition, we validated the combined hypoxia and tumor cell stemness status classifiers model using GSE31210. Patients were classified into 3 clusters: cluster a (N = 103), cluster b (N = 105) and cluster c (N = 13) (Fig. 1d). DFS of clusters was analyzed (Fig. 1e). Considering that the number of samples in cluster c was too small, we compared the prognosis of cluster a and b. The prognosis of cluster b was more satisfactory than cluster a (Fig. 1f). However, the stemness and hypoxia index of cluster a was higher than cluster b (Fig. 1d). These results were consistent with the analysis results of TCGA database, indicating that the combined hypoxia and tumor cell stemness status classifiers model was feasible. Hence, we mainly focus on cluster 3 and cluster 4 (in TCGA database) in the follow-up.

**Fig. 2:**
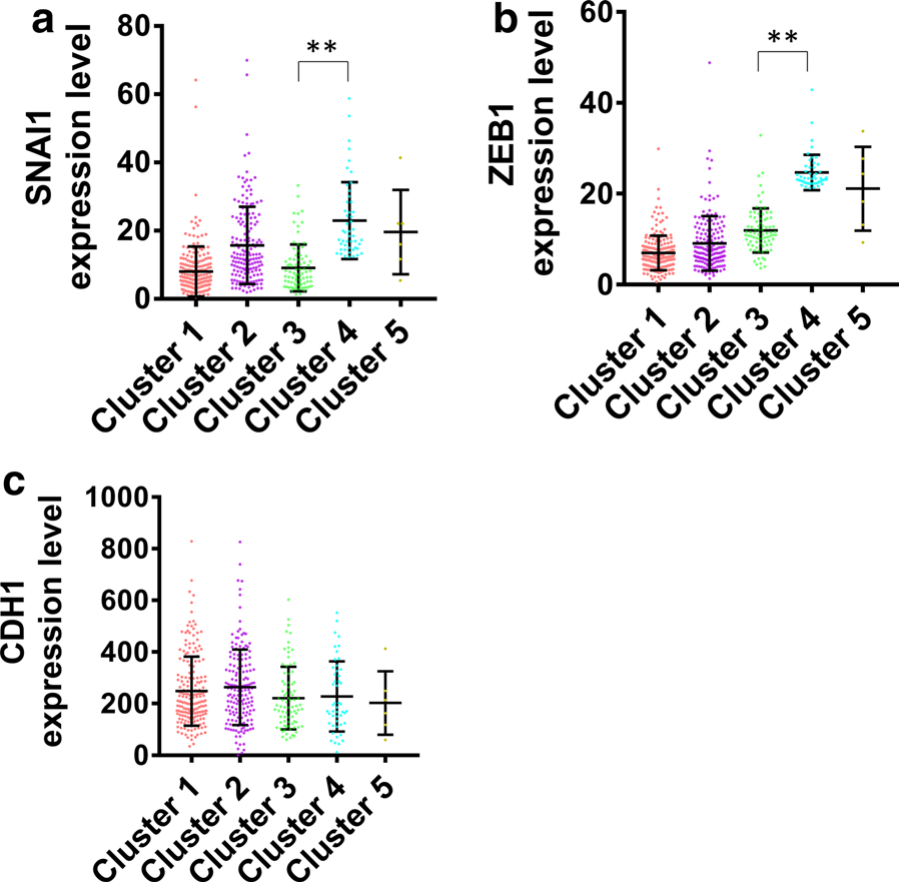
Expression analysis of EMT regulatory genes, SNAI1 (**a**), ZEB1 (**b**) and CDH1 (**c**), in different clusters. **P < 0.001

Furthermore, the expression of epithelial-mesenchymal transition (EMT) regulatory genes in different clusters were analyzed. The results suggested that the expression levels of SNAI1 and ZEB1 in cluster 4 were notably higher than those in cluster 3 (Fig. 2a, b). However, there was no significant difference in CDH1 expression between cluster 3 and cluster 4 (Fig. 2c).

### Regulation of stemness and hypoxia on mRNA expression

**Fig. 3:**
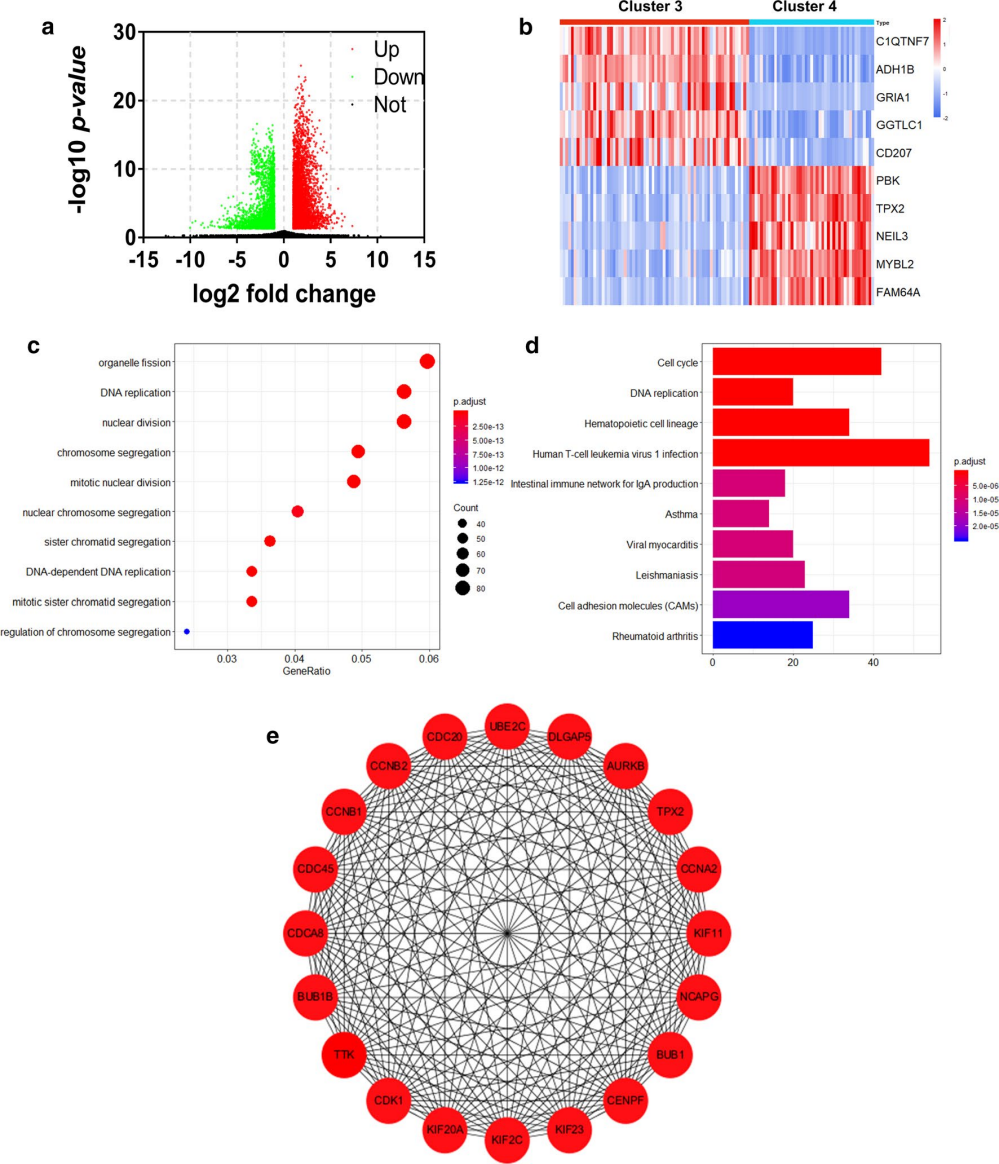
Identification and functional analysis of differential mRNAs between cluster 3 and cluster 4. **a** The volcano plot of differential expressed mRNAs in cluster 3 versus cluster 4. **b** Heatmap of expression pattern of the top 5 upregulated mRNAs and the top 5 downregulated mRNAs. **c** The top 10 GO terms of the differential mRNAs. **d** The top 10 KEGG pathways of the differential mRNAs. **e** Hub genes of the PPI network. GO, Gene Ontology; KEGG, Kyoto Encyclopedia of Genes and Genomes; PPI, protein–protein interaction

We found the transcript levels of 4341 mRNAs to be enhanced, and 2526 mRNAs to be repressed in cluster 3 compared with cluster 4 (Fig. 3a), implying that hypoxia and stemness obviously affected mRNAs expression at the genome-wide level. C1QTNF7, ADH1B, GRIA1, GGTLC1 and CD207 were the top 5 upregulated mRNAs (Fig. 3b). PBK, TPX2, NEIL3, MYBL2 and FAM64A were the top 5 downregulated mRNAs (Fig. 3b). Through GO analysis of 6867 DE mRNAs (including 4341 upregulated mRNAs and 2526 downregulated mRNAs), we found that the differential mRNAs were mainly involved in DNA replication, nuclear division and chromosome segregation (Fig. 3c). Congruently, KEGG analysis revealed that metabolic pathways of DE mRNAs enrichment were related to DNA replication and mitosis (Fig. 3d). The relationship of DE mRNAs was analyzed using the PPI network. Hub genes of PPI network were identified corresponded to MCC score and showed in Fig. 3e.

### Effects of hub genes expression on survival of patients with LUAD

**Fig. 4:**
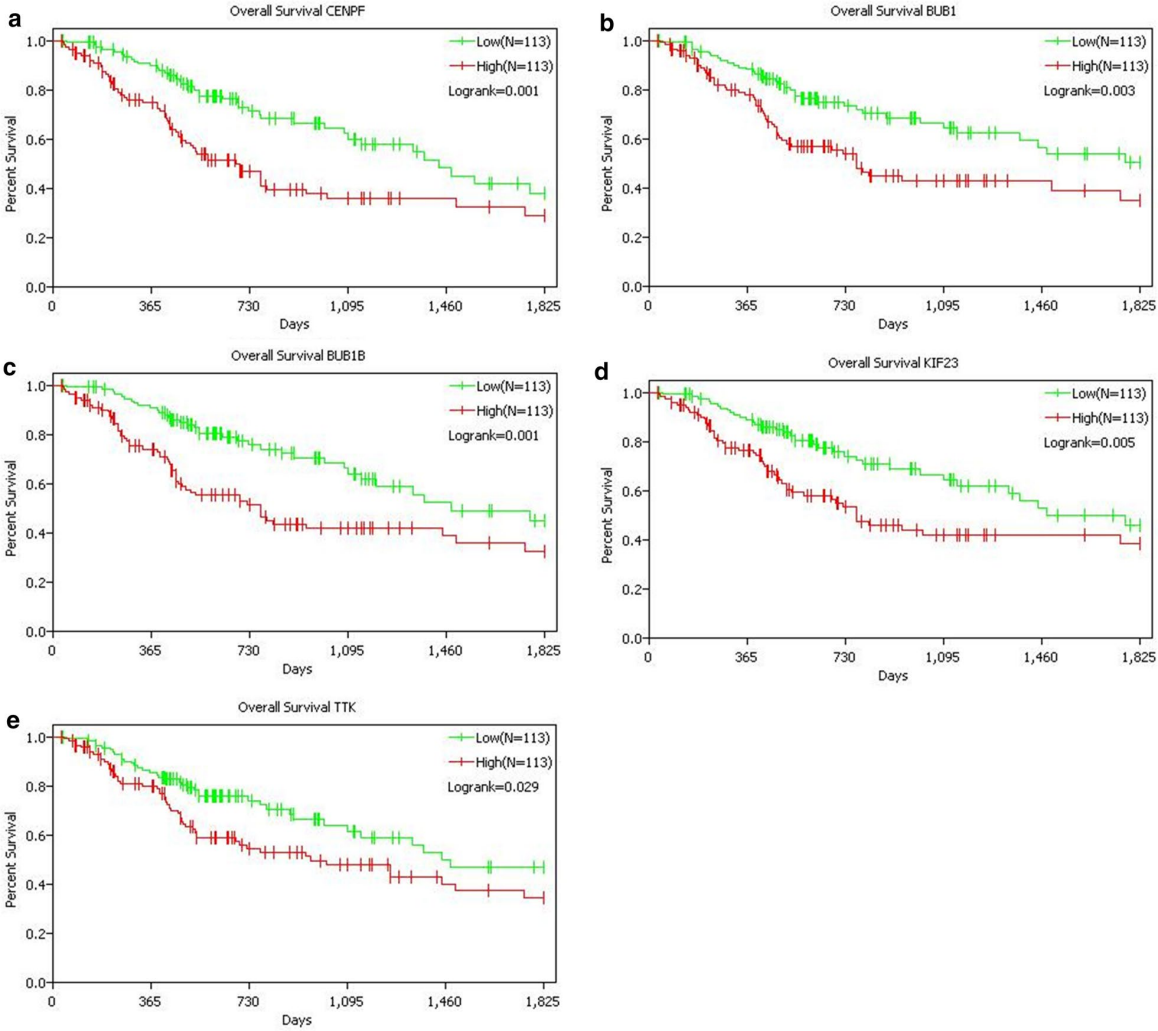
Prognostic analysis of CENFP (**a**), BUB1 (**b**), BUB1B (**c**), KIF23 (**d**) and TTK (**e**) in TCGA database. According to gene expression level, patients were divided into high expression group (N = 113) and low expression group (N = 113)

**Fig. 5:**
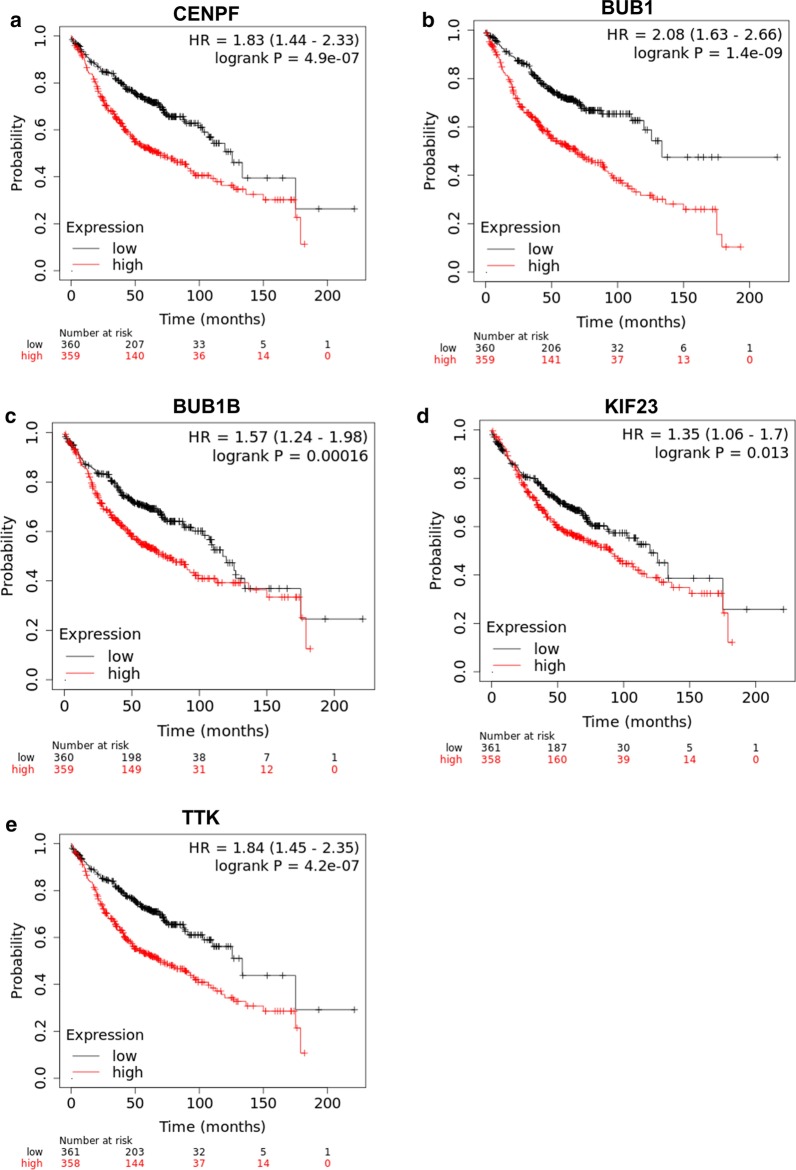
Prognostic analysis of CENFP (**a**), BUB1 (**b**), BUB1B (**c**), KIF23 (**d**) and TTK (**e**) in Kaplan Meier-plotter database

Kaplan–Meier curves were plotted to analyze the relationship between the expression of 20 hub genes and overall survival. In TCGA database, patients with high expression of CENFP, BUB1, BUB1B, KIF23 and TTK had poor prognosis, indicating that these gene expressions were beneficial to LUAD progression (Fig. 4). However, the expression of other hub genes had no significant effect on the prognosis of patients with LUAD. In Kaplan Meier-plotter database, the prognostic analysis results of the 20 hub genes were consistent with the results of the TCGA database (Fig. 5).

### Regulation of stemness and hypoxia on lncRNA and miRNA expression

**Fig. 6:**
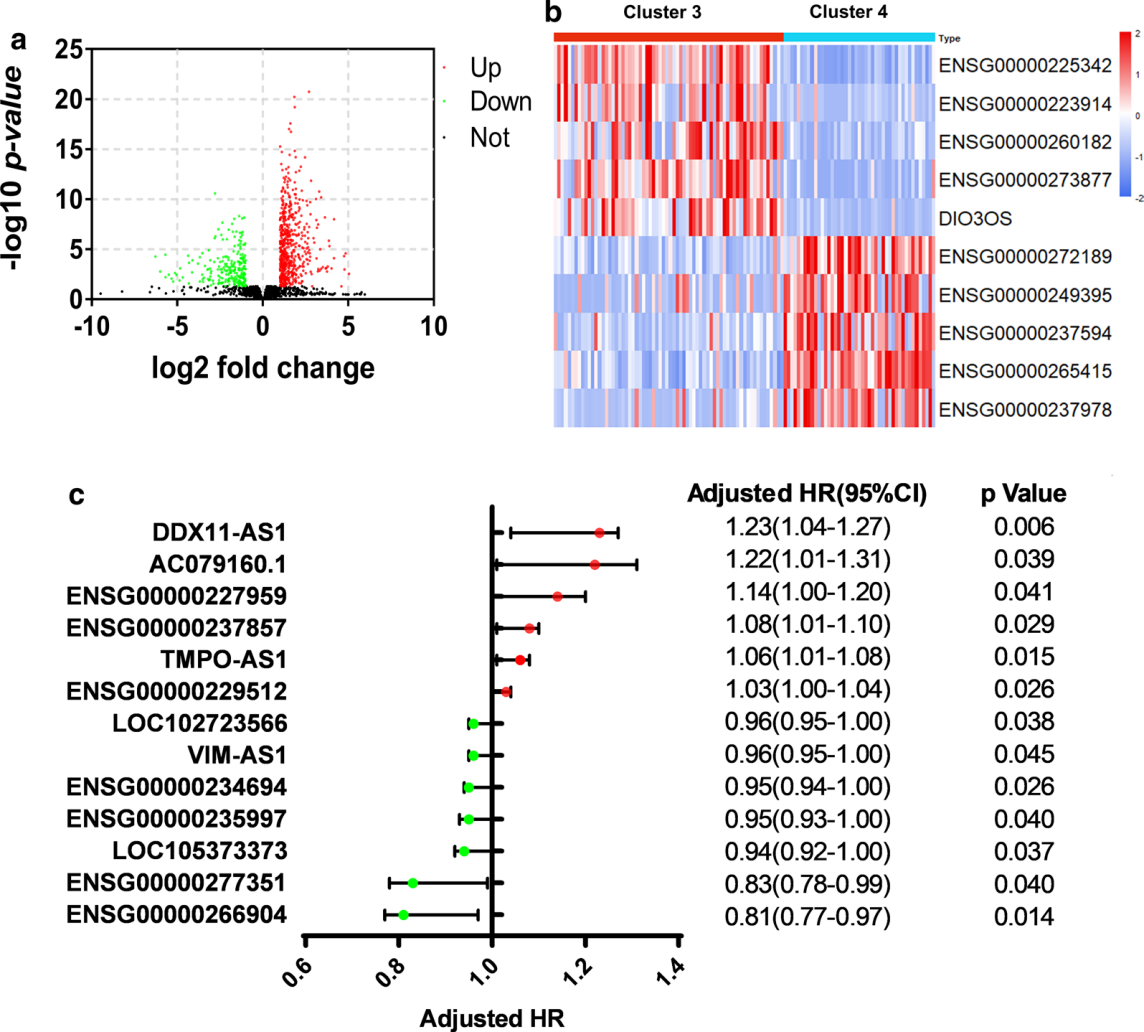
Identification and prognosis analysis of differential lncRNAs between cluster 3 and cluster 4. **a** Screening for up- and downregulated lncRNAs. **b** The top 5 up- and downregulated lncRNAs. **c** Cox proportional hazards regression analysis of the differential lncRNAs. HR, hazard ratio; CI, confidence interval

In order to further understand the effect of stemness and hypoxia on gene expression of LUAD cells, we screened the DE lncRNA between cluster 3 and cluster 4. There were a total of 807 DE lncRNAs, of which 564 lncRNAs were upregulated and 243 lncRNAs were downregulated (Fig. 6a). The DE lncRNAs were ranked according to FC. The top 5 upregulated lncRNAs and the top 5 downregulated lncRNAs were shown in Fig. 6b. Congruently, we estimated the HRs of the 807 DE lncRNAs. The DE lncRNAs which risk score could independently predict the overall survival of patients were displayed in Fig. 6c.

**Fig. 7:**
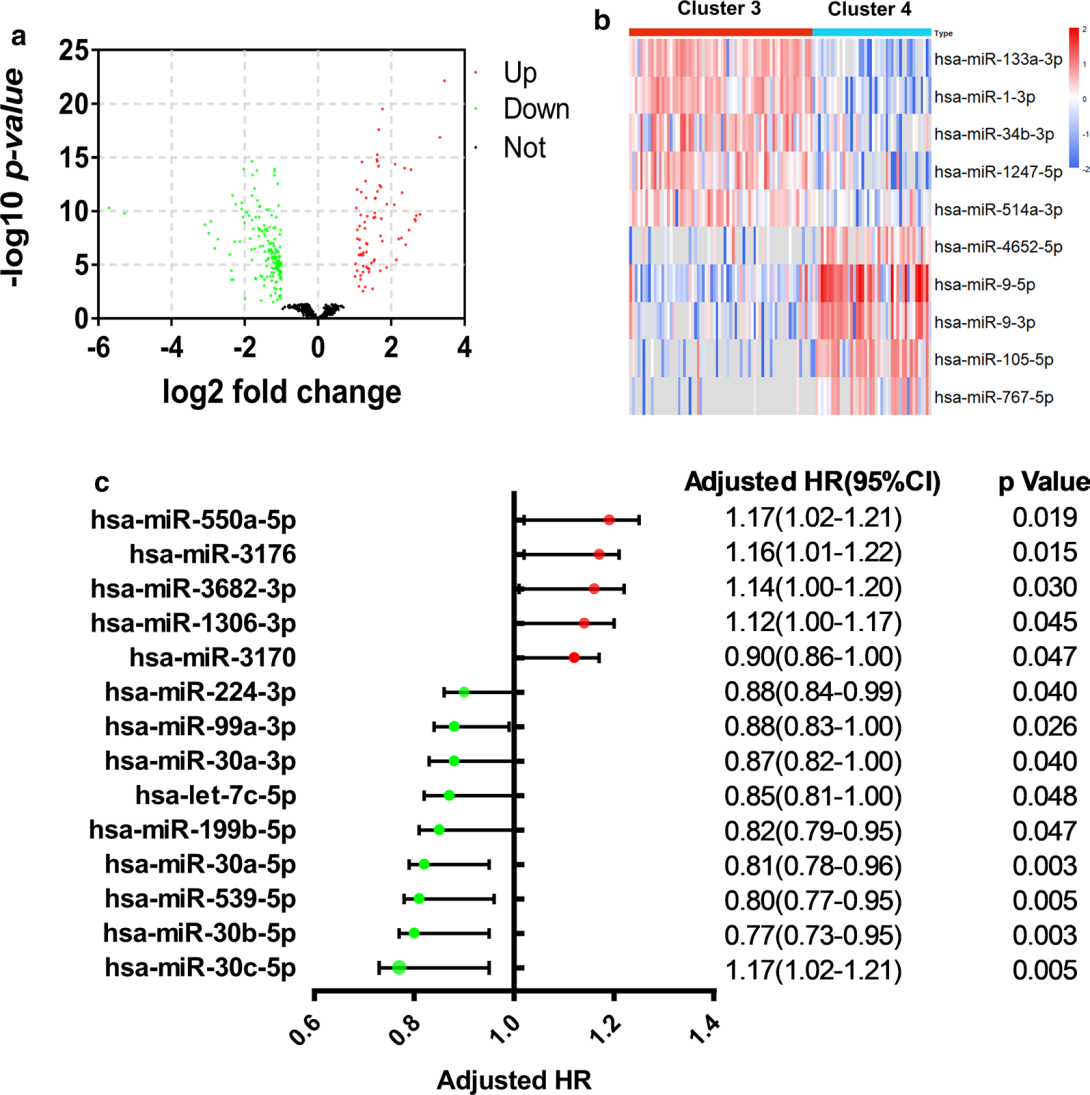
Screen and prognosis analysis of differential miRNAs in cluster 3 compared with cluster 4. **a** Screening for up- and downregulated miRNAs. **b** Heatmap of the top 5 up- and downregulated miRNAs. **c** Forest plot of Cox analysis for the differential miRNAs. HR, hazard ratio; CI, confidence interval

We also identified 77 upregulated miRNAs and 166 downregulated miRNAs in cluster 3 compared with cluster 4 (Fig. 7a). The top 5 upregulated miRNAs were has-miR-133a-3p, has-miR-1-3p, has-miR-34b-3p, has-miR-1247-5p and has-miR-514a-3p (Fig. 7b). The top 5 downregulated miRNAs were has-miR-4652-5p, has-miR-9-5p, has-miR-9-3p, has-miR-105-5p and has-miR-767-5p (Fig. 7b). In total, 14 DE miRNAs with significant influence on prognosis were obtained by Kaplan–Meier curve and log-rank test (Fig. 7c).

### CeRNA network analysis

**Fig. 8:**
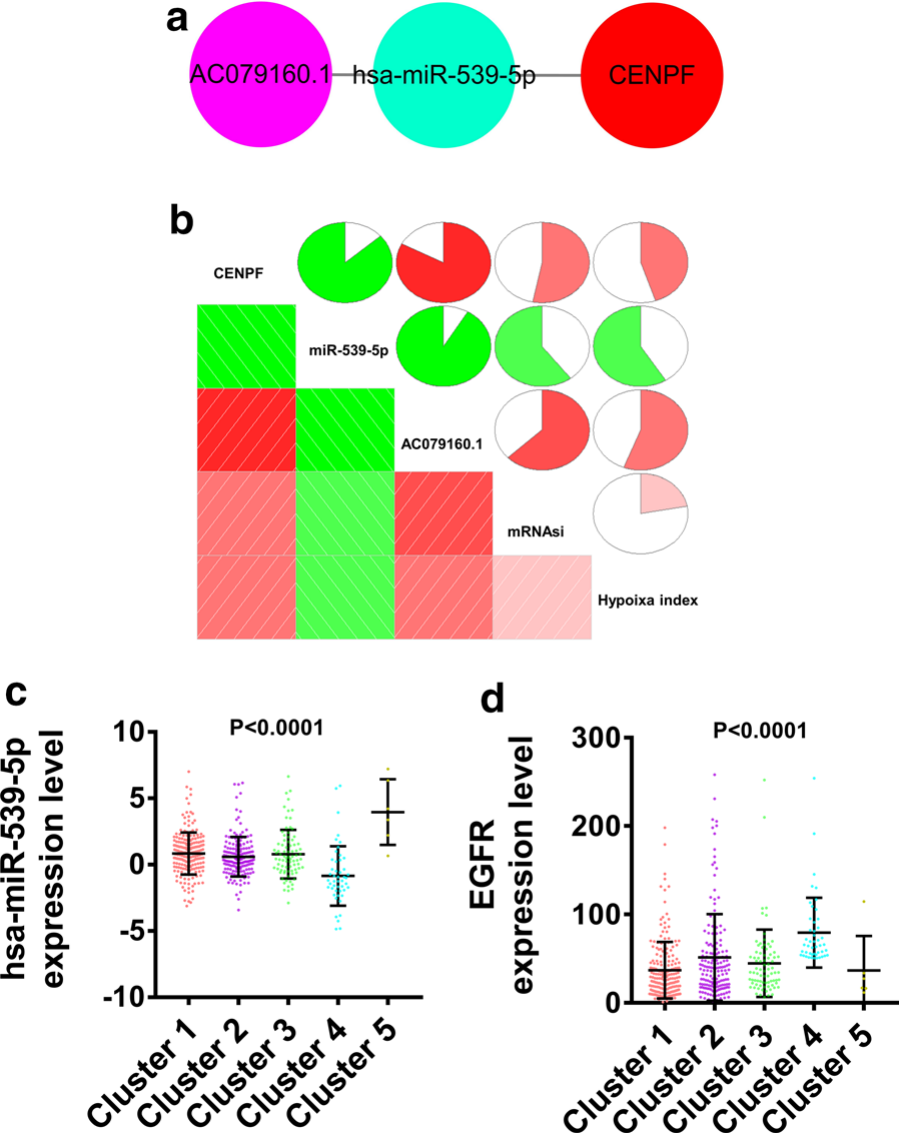
ceRNA network analysis of hub genes, differential lncRNA and miRNA with significant influence on prognosis. **a** CeRNA network. Red, differentially expressed mRNA. Purple, differentially expressed lncRNA. Blue, differentially expressed miRNA. **b** Correlation analysis of node expression in ceRNA network with hypoxia and stemness in LUAD. Red, positive correlation. Green, negative correlation. mRNAsi, mRNA expression-based stemness index. **c**, **d** The expression of miR-539-5p (**c**) and EGFR (**d**) in different clusters

The ceRNA network was constructed by using the hub genes, DE lncRNA and DE miRNA, which significantly affected prognosis. LncRNA-miRNA-mRNA ceRNA network was based on the following principles: lncRNA directly interacts by invoking miRNA sponge to regulate mRNA activity, and the expression correlation among lncRNA, miRNA and mRNA. Finally, we obtained a ceRNA network, “AC079160.1-miR-539-5p-CENPF” (Fig. 8a). The correlation relationship among the expression levels of CENPF, AC079160.1, miR-539-5p, tumor hypoxia and stemness index was evaluated. The expression level of CENPF was positively correlated with AC079160.1 expression, hypoxia and stemness index, but negatively correlated with miR-539-5p expression (Fig. 8b). miR-539-5p expression level was negatively correlated with hypoxia and stemness index (Fig. 8b). AC079160.1 expression was positively correlated with hypoxia and stemness index (Fig. 8b). Subsequently, we analyzed the expression of miR-539-5p and EGFR in different clusters. As expected, the expression of miR-539-5p in cluster 4 was significantly lower than other clusters, while the expression of EGFR in cluster 4 was notably higher than other clusters (Fig. 8c, d).

**Fig. 9:**
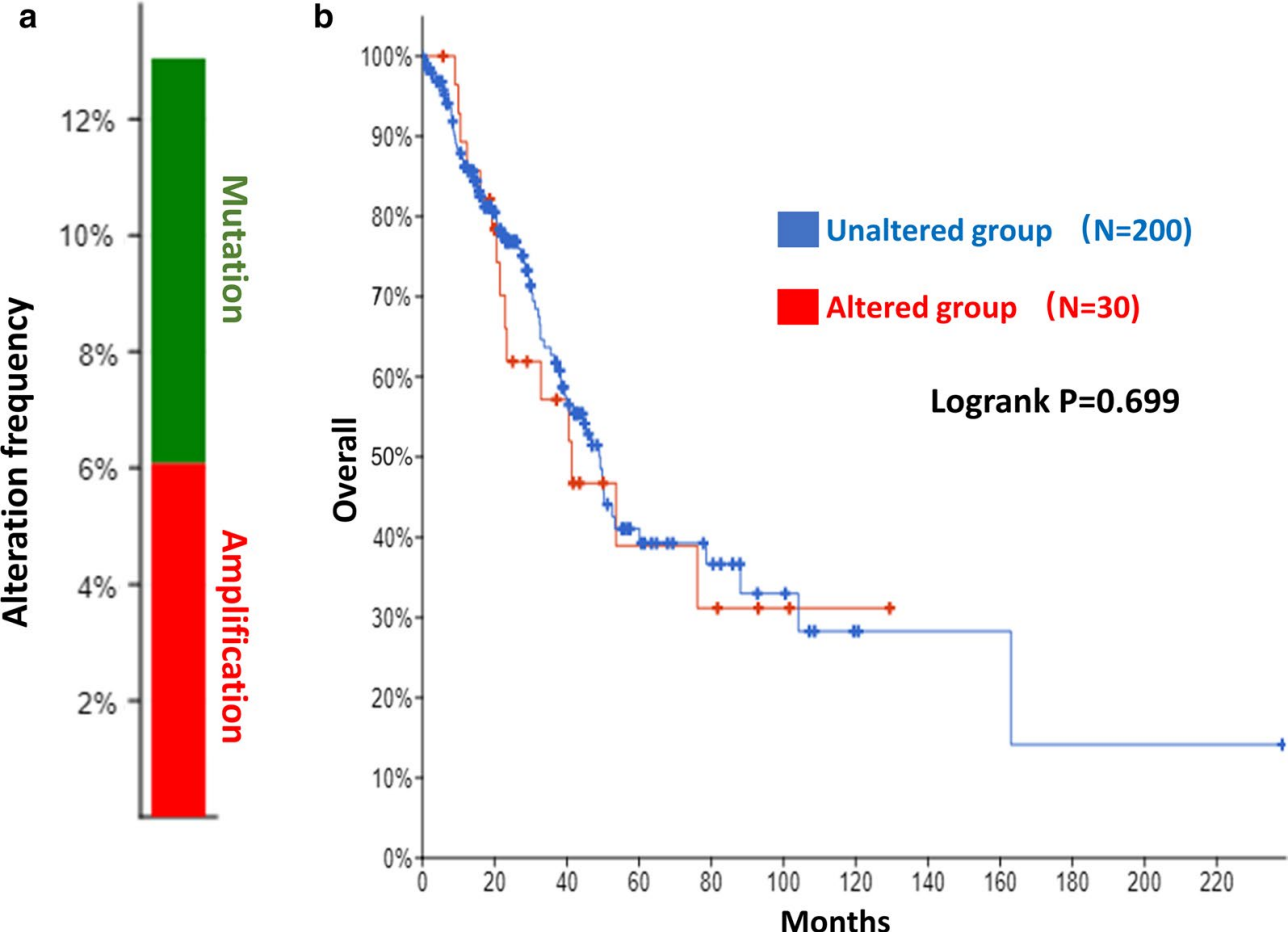
Prognostic analysis of the copy number variation of CENPF. **a** Alteration frequency of CENPF in LUAD. **b** Overall survival of patients in unaltered group (N = 200) and altered group (N = 30)

Since CENPF is one of the key DE genes, we analyzed the copy number variation of CENPF using cBioPortal database. CENPF was altered in 13% (including 6% amplification and 7% mutation) of LUAD patients/samples (Fig. 9a). However, further survival analysis showed that the copy number variation of CENPF does not affect the prognosis of patients (Fig. 9b).

## Discussion

In recent years, significant progress has been made in the treatment of LUAD. The survival rate of patients with LUAD has improved, but the efficacy of its treatment is still not satisfactory. The 5-year overall survival rate of LUAD is only 15% [4]. Therefore, exploring new treatment strategies for LUAD have become the focus of current research. Intratumoral hypoxia and tumor cell stemness are associated with patient outcome in various solid tumor [5, 6, 10]. However, these factors have not yet considered for treatment selection in LUAD due to lack of validated biomarkers. This study obtained expression data of LUAD from TCGA database, and explored a combined hypoxia and tumor cell stemness status classifiers model. Our study showed that LUAD patients with high hypoxia and stemness index have a poor prognosis, while those with low hypoxia and stemness index have a better prognosis. The classifiers model explored in this study was validated using GSE31210, and the results were consistent with the analysis results of TCGA. This reflected the rationality and usability of the classification model. Hypoixa and stemness are important regulators of EMT. E-cadherin, encoded by CDH1, is a key factor in inhibiting EMT [28]. SNAI1 and ZEB1 are important transcription factors that promote the progress of EMT, and play a key role in the occurrence and development of a variety of cancers [29]. Our results indicated that although the difference of CDH1 expression in each cluster was not obvious, the expression of SNAI1 and ZEB1 in the high hypoxia and stemness index cluster (cluster 4) was significantly upregulated. Therefore, systematic analysis of the relationship between tumor hypoxia and stemness can provide a more targeted research area and a new perspective to reveal the underlying mechanism of cancer.

Hypoxia affects stem cell phenotype through multiple signaling pathways [30–32]. In this study, we obtained 6867 protein-coding genes related to hypoxia and tumor stemness. Annotation and functional analysis showed that these genes were mainly involved in cell cycle and DNA replication. These results suggested that hypoxia-induced tumor stem cells have potent proliferation ability. Studies have shown that hypoxia can make lung cancer, glioma and prostate cancer cells to express stem cell characteristics, maintain their undifferentiated state, or increase the number of tumor stem cells [33, 34]. To further screen the key genes in the hypoxia associated tumor stemness during LUAD progression, we constructed a PPI network and performed prognostic analysis. CENPF, BUB1, BUB1B, KIF23 and TTK were identified as the key potential genes affecting hypoxia associated tumor stemness in this study. CENPF is a protein associated with the centromere-kinetochore complex [35]. Studies have demonstrated that breast cancer patients with high expression of CENPF in tumor tissue are more prone to bone metastasis [36]. The process of tumor bone metastasis is usually accompanied by the enhancement of hypoxia and stemness of tumor cells [37]. BUB1 and BUB1B are key components of the mitotic checkpoint complex. Abnormal expression or mutation of BUB1 or BUB1B can lead to aneuploidy. Considering that aneuploidy is common in many types of cancer, it is believed that an uncontrolled spindle checkpoint leads to the development of cancer [38]. KIF23 plays an important role in the process of mitosis, which is closely related to the occurrence and development of liver cancer [39]. In addition, the expression level of TTK is significantly increased in various malignant tumors, and its high expression is closely related to a poor prognosis [40]. However, we have not found any research on the progress of LUAD regulated by CENPF, BUB1, BUB1B, KIF23 or TTK. The mechanism by which these genes affect the hypoxia and stemness of LUAD needs further study.

The mechanism regulating the cancer occurrence and development is complex. The role of a single gene or pathway has been very limited. The systematic construction and analysis of ceRNA networks can provide more targeted perspectives to reveal the underlying mechanisms of cancer [41]. In this study, a total of 807 DE lncRNAs and 243 DE miRNAs were screened. Subsequently, the DE lncRNA related to DE miRNA was screened through the bioinformatics database, and the ceRNA network related to hypoxia induced tumor cell stemness was constructed. We identified the ceRNA network with CENPF, AC079160.1 and has-miR-539-5p as key nodes. The role of miR-539 in several human cancers has been revealed, and recent studies displayed its contradictory role in cancer [42–44]. Research shows that miR-539 inhibits lung cancer cell proliferation and metastasis by directly targeting EGFR [45]. The EGFR-dependent signaling pathways are closely related to the tumor hypoxic microenvironment. Generally, tumor cells are accompanied by high expression of EGFR under hypoxic conditions [46]. In this study, we found that EGFR was highly expressed in the high hypoxia and stemness index cluster, while miR-539-5p was lowly expressed. These results were consistent with the literature reports, proving the reliability of our research [45, 46]. These results indicated that miR-539-5p may be an important inhibitor of hypoxia-induced stemness of tumor cells. As a "sponge" of miRNA, lncRNA can inhibit miRNA activity. The dysregulation of lncRNA expression can lead to different ceRNA-mediated mechanisms of action, thus playing the role of oncogenes or tumor suppressor genes [47, 48]. Some lncRNAs have been reported to be involved in LUAD metastasis, which are closely related to the poor prognosis of LUAD [49, 50]. In the "AC079160.1-has-miR-539-5p-CENPF" axis, AC079160.1 as the ceRNA of has-miR-539-5p participated in the regulation of hypoxia-induced stemness of tumor cells, and affected the progress of LUAD. At present, we have not found any studies on the regulation of LUAD by AC079160.1. This study analyzed the regulatory target of AC079160.1, which provided a new research idea for understanding its mechanism of action, which is worthy of further verification by experiment. Moreover, we analyzed the alteration frequency of CENPF in LUAD, and its influence on the prognosis. We found that the variation of CENPF copy number had no significant effect on the prognosis of LUAD. These results further suggested that the ceRNA network is an important regulatory mechanism of CENPF.

In conclusion, we found that CENPF, BUB1, BUB1B, KIF23 and TTK were potential key genes involved in regulating hypoxia induced tumor cell stemness. Additionally, we found that the "AC079160.1-miR-539-5p-CENPF" axis was an important regulatory pathway in hypoxia-induced tumor cell stemness.

## Data Availability

The datasets used and/or analyzed during the present study are available from the corresponding author (Email: tangjf1969@163.com) on reasonable request.
